# Annular Erythema in a Nine-Year-Old Girl: A Rare Type of Psoriasis

**DOI:** 10.7759/cureus.89836

**Published:** 2025-08-11

**Authors:** Maho Matsuo, Hiroaki Iwata

**Affiliations:** 1 Dermatology, Gifu University Graduate School of Medicine, Gifu, JPN

**Keywords:** annular erythema, biologics, il-17a, pediatric, psoriasis

## Abstract

Annular erythema is a nonspecific clinical sign arising from various conditions, including infections, cutaneous lymphomas, connective tissue diseases, and inflammatory dermatoses. Psoriasis vulgaris typically presents as sharply demarcated erythematous plaques with silvery scales; however, annular forms are rare and may complicate diagnosis. We report a nine-year-old girl with an annular erythematous lesion on the anterior chest that gradually expanded over two months, spreading to the extremities and trunk with mild pruritus. Despite suspicion of tinea corporis, repeated fungal tests were negative, and topical corticosteroids were ineffective. Skin biopsy showed marked hyperkeratosis, loss of the granular layer with parakeratosis, and subcorneal Munro's microabscesses; no fungal elements were found. Laboratory tests and imaging were unremarkable, and genetic screening for IL36RN and CARD14 found no pathogenic variants. A diagnosis of psoriasis vulgaris was made with a Psoriasis Area and Severity Index (PASI) score of 7.8. After failed topical treatment, systemic secukinumab therapy was initiated, leading to complete lesion resolution within two months. This case emphasizes the importance of considering psoriasis in the differential diagnosis of pediatric annular erythema and demonstrates the value of histopathology and biologics in managing atypical pediatric psoriasis.

## Introduction

In the differential diagnosis of erythema annulare, clinicians must evaluate a wide array of potential etiologies, encompassing infectious agents, cutaneous lymphoproliferative disorders, connective tissue pathologies, and diverse inflammatory conditions [1]. Psoriasis is a chronic papulosquamous inflammatory skin disease that can affect individuals at any age, but it is more likely to occur in their 40s to 50s [2,3]. Psoriasis vulgaris characteristically manifests as confluent erythematous plaques surmounted by silvery-white scales [3]. Nevertheless, it may occasionally assume an annular morphology, a presentation partially ascribed to the Koebner phenomenon [1]. Annular lesions are especially indicative of a rare subtype termed annular pustular psoriasis, which is more frequently observed in pediatric and young adult populations. Especially, it is well known that pustular psoriasis demonstrated annular phenotypes [4,5]. In pustular subtypes, erythematous pustules frequently coalesce to form annular configurations, with such patterns being more prevalent in pediatric populations than in adults [6,7]. Distinctive hallmarks of classic psoriasis often remain discernible at the peripheral margins of the annular lesions. Consequently, through meticulous clinical assessment and the application of appropriate diagnostic modalities, clinicians can achieve an accurate diagnosis and promptly initiate optimal therapeutic interventions. 

## Case presentation

**Figure 1:**
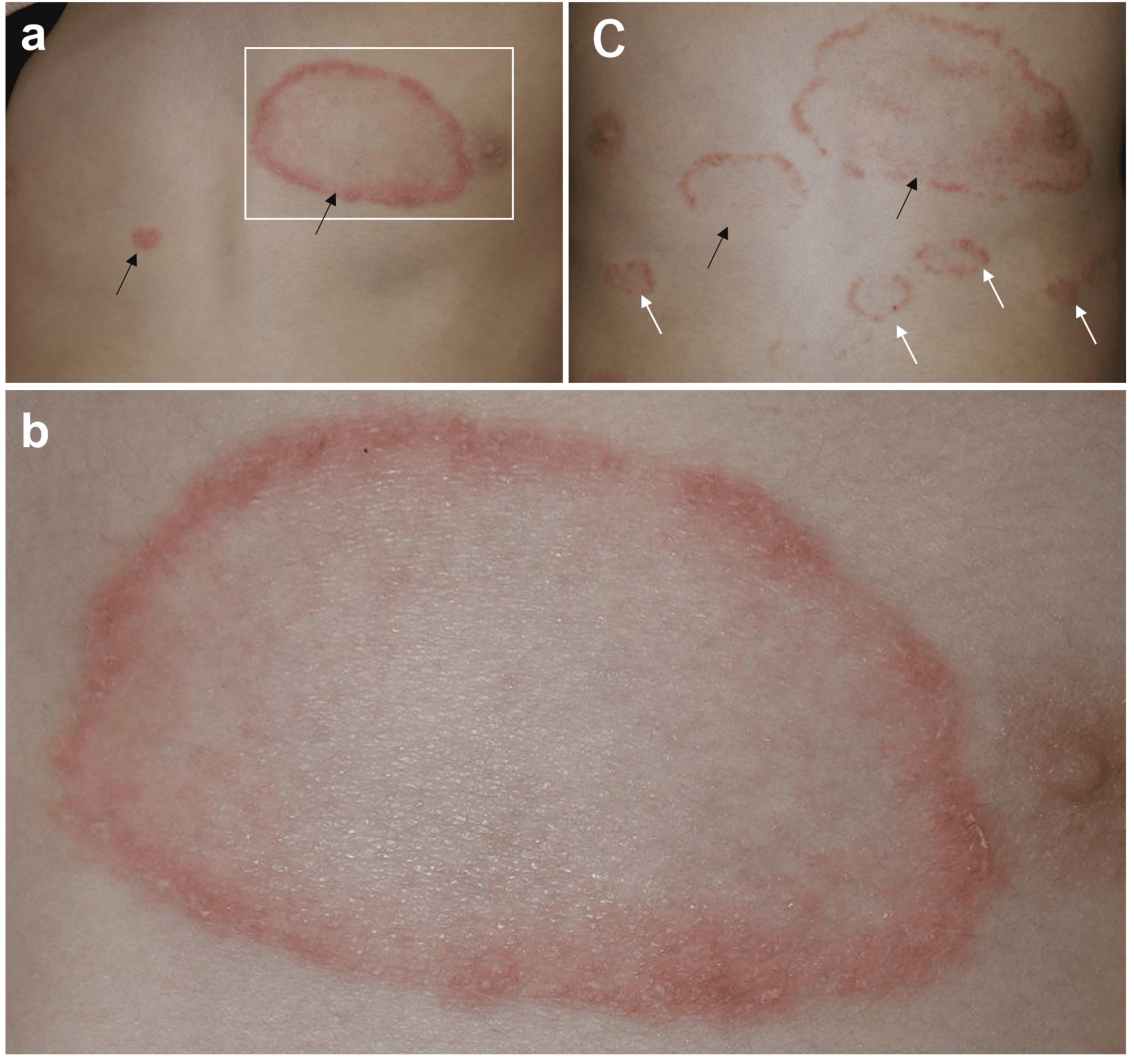
Clinical manifestations of annular psoriasis. (a) Annular erythematous lesions accompanied by elevated and hyperkeratotic scales on the margin are observed, localized to the anterior chest at the initial visit. (b) Close observation of the square in (a). (c) Lesions progressively spread to the trunk and extremities despite topical corticosteroids and vitamin D3 analogs. Black arrows indicate expanding lesions, and white arrows indicate new lesions.

A nine-year-old girl developed erythema on the anterior chest two months earlier, which progressively and centrifugally expanded in an annular pattern. She had no family history of psoriasis or pustular psoriasis. Her medical history was notable for vitiligo, but no other antibody-mediated autoimmune disorders, including connective tissue diseases. In addition, she had a peanut allergy, but neither chronic sinusitis nor repeated pharyngitis. The erythematous lesions subsequently spread to the extremities, scalp, buttocks, and trunk, accompanied by mild pruritus. She had neither arthralgia nor nail involvement. She was initially suspected of having tinea corporis, but repeated potassium hydroxide (KOH) examinations yielded negative results. Although she was treated with topical corticosteroids, there was no clinical improvement. Rather, the annular lesions expanded outward, and new lesions appeared, prompting referral to our department. Clinical examination reveals annular erythematous lesions associated with fine scales localized to the anterior chest (Figure 1a). During the clinical course, no pustules were observed on the erythematous lesions.

**Figure 2:**
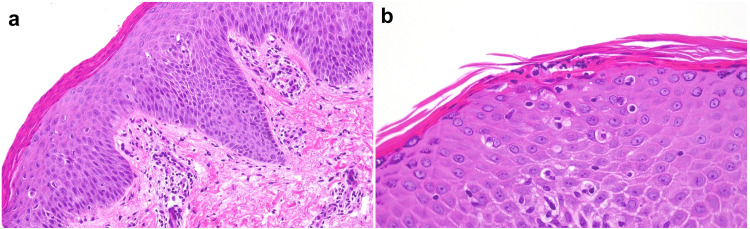
Histopathological findings in annular psoriasis. (a) Skin biopsy from the border of the annular lesion demonstrating acanthosis, marked hyperkeratosis and loss of the granular cell layer with associated parakeratosis. (b) Presence of Munro’s microabscesses.

**Figure 3:**
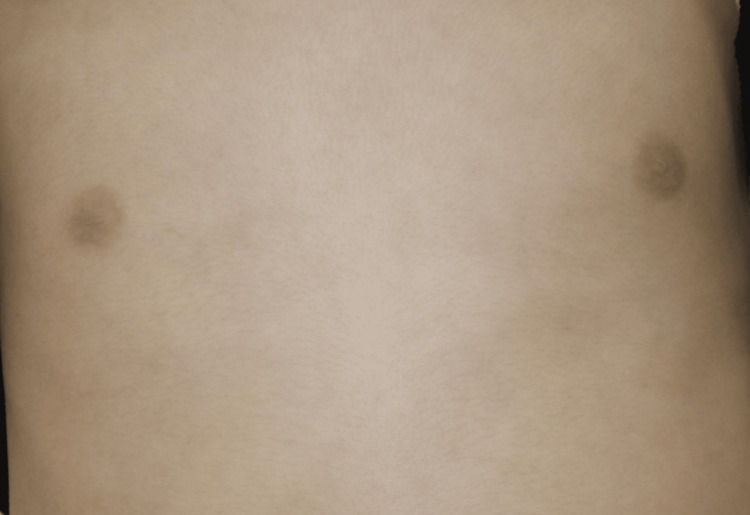
Two months after treatment with secukinumab. Complete resolution of the skin lesions after two months of treatment with secukinumab (75 mg/body).

Skin biopsy from the border of the annular lesion revealed marked hyperkeratosis, loss of the granular cell layer with associated parakeratosis (Figure 2a), and Munro’s microabscesses (Figure 2b). No fungal elements were detected in the stratum corneum. In the upper dermis, perivascular lymphocyte infiltrates were present without atypical lymphocytes. We did not find typical coat-sleave-like infiltrates, suggesting erythema annulare centrifugum. Based on these findings, particularly the characteristic histological features, a definitive diagnosis of psoriasis vulgaris was established. The Psoriasis Area and Severity Index (PASI) was 7.8. Blood tests and CT revealed no significant findings. No pathogenic variants were identified in IL36RN or CARD14. Topical therapy with corticosteroid and vitamin D3 analogs was initiated, but there was no clinical improvement; instead, the lesions progressively worsened (Figure 1b). Consequently, systemic therapy with secukinumab, an anti-IL-17A monoclonal antibody, was introduced (body weight 21 kg, 75 mg subcutaneous injection at zero, one, two, three, and four weeks, followed by a dose every four weeks), leading to complete resolution of the lesions within two months (Figure 3).

There are no relapses for the half-year observations. In this case, we considered other systemic therapies, such as cyclosporine or phototherapy, but finally administered secukinumab with deep informed consent. In Japan, systemic phototherapy is not recommended for those under 10 years old. In addition, the patient and parent did not want to have cyclosporine due to side effects, such as hirsutism or the risk of carcinogenesis.

## Discussion

Psoriasis is a chronic, immune-mediated inflammatory skin disease that follows a relapsing and remitting course [3,8]. It is influenced by a complex interplay of genetic susceptibility [9], such as IL36RN or CARD14, and environmental factors. Although psoriasis typically manifests in middle age, pediatric cases are relatively rare [2,3,8].

Clinically, psoriasis exhibits heterogeneous phenotypes, including plaque, guttate, and pustular types. The most common form is psoriasis vulgaris, characterized by well-demarcated erythematous plaques covered with adherent, scaly surfaces that may evolve into characteristic silvery scaling. Psoriasis vulgaris rarely presents an annular (ring-like) configuration, but this pattern should be considered in the differential diagnosis of erythema annulare [1,6]. Annular lesions are particularly characteristic of a rare subtype known as annular pustular psoriasis, which is more often seen in children and young adults [6,7]. This form presents as slowly expanding, ring-shaped plaques with red, scaly borders and pustules at the edges. Compared to generalized pustular psoriasis, the annular variant shows a milder clinical course but may recur chronically. In the present case, although the patient did not exhibit visible pustules, histopathological examination clearly revealed subcorneal microabscesses, supporting a diagnosis of psoriasis.

When encountering annular skin lesions, a broad range of differential diagnoses should be considered. The most common is tinea corporis, which typically presents with erythema, peripheral scaling, and central clearing. Other conditions to consider include erythema annulare centrifugum; mycosis fungoides, the most common cutaneous T-cell lymphoma; cutaneous lupus erythematosus; secondary syphilis; and seborrheic dermatitis. In our case, these possibilities were excluded based on negative fungal examinations and characteristic histopathological findings.

Psoriasis is not a simple skin disorder, but it is now recognized as a systemic inflammatory condition. It is strongly associated with several comorbidities, including psoriatic arthritis, metabolic syndrome, diabetes, and cardiovascular disease [10,11]. Therefore, appropriate control of skin inflammation is important not only for dermatological improvement but also for preventing systemic complications. Recent studies have elucidated the central role of tumor necrosis factor-alpha (TNF-α), IL-23, and IL-17 in the pathogenesis of psoriasis [8]. Targeting these cytokines has led to significant improvements in both skin symptoms and systemic inflammation. Early therapeutic intervention is essential to reduce the risk of long-term complications, including physical and psychological burdens. Biologics have become a key therapeutic option for managing moderate to severe psoriasis. However, treatment options for pediatric psoriasis are limited, mainly due to a lack of clinical trial data in this population [12,13]. In this case, we selected secukinumab, an anti-IL-17A monoclonal antibody, which is approved for pediatric use and covered by insurance. A recent randomized controlled trial showed that approximately 80% of pediatric patients with moderate to severe plaque psoriasis achieved PASI75, indicating a 75% reduction in disease severity, within 12 weeks of treatment [13].

## Conclusions

In conclusion, annular erythema can be a clinical manifestation of numerous dermatologic conditions, and although uncommon, psoriasis should be considered within the differential diagnosis. Annular psoriasis may obscure the diagnostic process, especially in pediatric or atypical cases. However, hallmark clinical and histopathological features of classical psoriasis often remain at the lesion periphery. Through meticulous clinical assessment and the application of appropriate diagnostic modalities, clinicians can establish an accurate diagnosis and deliver targeted therapeutic interventions. So far, the patient has no relapse, but we are carefully observing due to a rare case. 
